# Reducing the risk of impaired bone apposition to titanium screws with the use of fibroblast growth factor-2−apatite composite layer coating

**DOI:** 10.1186/s13018-016-0501-z

**Published:** 2017-01-05

**Authors:** Kengo Fujii, Atsuo Ito, Hirotaka Mutsuzaki, Shinji Murai, Yu Sogo, Yuki Hara, Masashi Yamazaki

**Affiliations:** 1 Department of Orthopaedic Surgery, Faculty of Medicine, University of Tsukuba, 1-1-1 Tennodai, Tsukuba, Ibaraki 305-8575 Japan; 2 Health Research Institute, National Institute of Advanced Industrial Science and Technology (AIST), Central 6, 1-1-1 Higashi, Tsukuba, Ibaraki 305-8566 Japan; 3 Department of Orthopaedic Surgery, Ibaraki Prefectural University of Health Sciences, 4669-2 Ami Ami-machi, Inashiki-gun, Ibaraki 300-0394 Japan

**Keywords:** Fibroblast growth factor-2 (FGF-2), Apatite, Coating, Titanium screw, Bone formation, Weibull plot analysis, Impaired bone formation risk

## Abstract

**Background:**

Loosening of screws is a common problem in orthopedic and maxillofacial surgery. Modifying the implant surface to improve the mechanical strength of screws has been tried and reported. We developed screws coated with fibroblast growth factor-2 (FGF-2)−apatite composite layers. We then showed, in a percutaneous external fixation model, that this composite layer had the ability to hold and release FGF-2 slowly, thereby reducing the risk of pin tract infection of the percutaneous external fixation. The objective of the current study was to clarify the effect of FGF-2−apatite composite layers on titanium screws on bone formation around the screw.

**Methods:**

We analyzed samples of previously performed animal experiments. The screws were coated with FGF-2−apatite composite layers by immersing them in supersaturated calcium phosphate solutions containing FGF-2. Then, the uncoated, apatite-coated, and FGF-2−apatite composite layer-coated screws were implanted percutaneously in rabbits. Finally, using inflammation-free histological sections, we histomorphometrically assessed them for the presence of bone formation. Weibull plot analysis was then applied to the data.

**Results:**

On average, screws coated with FGF-2−apatite composite layers showed a significantly higher bone apposition rate than the uncoated or apatite-coated screws. Although the difference in the average bone apposition rate was small, the FGF-2−apatite composite layers produced a significant, marked reduction in the incidence of impaired bone formation around the screw compared with the incidence in the absence of FGF-2 (uncoated and apatite-coated screws). The probability of resulting in a bone apposition rate equal to or less than 63.75%, for example, is 3.5 × 10^-4^ for screws coated with the FGF-2−apatite composite layers versus 0.05 for screws in the absence of FGF-2.

**Conclusions:**

FGF-2-apatite composite layer coating significantly reduced the risk of impaired bone apposition to the screw. Thus, it is feasible to use titanium screws coated with FGF-2−apatite composite layers as internal fixation screws to decrease the risk of loosening.

## Background

Loosening of screws is a severe clinical problem in orthopedic and maxillofacial surgery, and it could be exacerbated in patients with compromised bone quality [1–3]. Screw loosening leads to unfavorable clinical results, including incomplete healing of a bone fracture and delayed union of spinal fusion. To prevent screw loosening, a variety of improvements and developments have been achieved in the surgical techniques for screw insertion and in the screw materials, design, and surface [4, 5].

The improved screw surface is considered a promising solution strategy. Such improvement includes surface modifications to enhance biocompatibility and osteoconductivity with the use of calcium phosphates, TiO_2_−strontium−CaSiO_3_−biopolymer composite, acid-etching, zinc-modified Ca−Si ceramic, and CaTiSiO_5_ ceramic [5–12]. Moreover, biologically active molecules such as fibroblast growth factor-2 (FGF-2), bone morphogenetic proteins (BMP), collagen, fibronectin, 1,25-vitamin D_3_, semaphorin 3A, and bisphosphonate are combined with the surface or incorporated into osteoconductive coatings [11, 13–20].

Screws coated with FGF-2−apatite composite layers are promising. FGF-2 regulates the proliferation and differentiation of osteoblasts and fibroblasts, and it promotes bone formation in appropriate doses [21–24]. Apatite is an osteoconductive material that is widely used in orthopedic and maxillofacial surgery [5–7]. The FGF-2−apatite composite layers form during immersion of the screws in infusion fluid-based supersaturated calcium phosphate solutions containing FGF-2 [13, 25, 26]. FGF-2 co-precipitates with calcium phosphate and consequently can be incorporated into the apatite layer. The FGF-2-apatite composite layer retains FGF-2 molecules, which are detectable by the enzyme-linked immunosorbent assay, in the layer for 16 days in Dulbecco’s modified Eagle’s medium at 37 °C [23]. Titanium external fixation screws coated with an FGF-2−apatite composite layer showed significantly higher bone−screw interface strength than those without the composite layer, owing to the osteoconductive nature of apatite in a percutaneous implantation model [25]. The FGF-2−apatite composite layer reduced the screw-tract infection rate through enhanced skin tissue healing [25]. Screw-tract infection greatly contributes to titanium screw loosening because an infection at the screw−skin interface evokes inflammation that, in turn, causes impaired bone formation around, and bone apposition to, the screws. In the case of internal fixation, however, infection-mediated loosening is hardly plausible because the entire fixation screw is present underneath the skin. Loosening of the internal screws depends solely on bone formation and apposition. Although we showed in a previous study that an FGF-2−apatite composite layer with a low dose of FGF-2 resulted in increased bone formation over an apatite layer in a rat cranial bone defect model, this result is insufficiently relevant to the feasibility of internal fixation screws coated with an FGF-2−apatite composite layer [23].

The purpose of the present study was to evaluate the feasibility of applying the FGF-2−apatite composite layer to internal fixation screws by evaluating bone formation around the screws. We retrospectively analyzed bone formation around the screws on all of the inflammation-free histological sections obtained previously by percutaneous implantation of titanium external fixation screws coated with FGF-2−apatite composite layers. Bone apposition to the screw and the comparative risk of impaired bone formation were assessed.

The risk of impaired bone formation was evaluated using the Weibull plot analysis, which is used to analyze the lifetime, failure probability, and/or reliability of industrial products [27]. In the present study, impaired bone formation around the screw was regarded as *failure* of the treatment. The Weibull plot analysis was therefore used to determine the probability of failure. Theoretically, the Weibull plot provides a straight line. The greater the slope of the line, the more constant is the outcome of the treatment and the lower the probability of failure.

## Methods

### Preparation of implants

The screws employed in the present study were commercially available. Composed of gamma ray-sterilized titanium (4.0 mm diameter, 30 mm long), they were cancellous screws (#407-030; Synthes Inc., West Chester, PA, USA) with an anodically oxidized surface. Under sterile conditions, the screws were immersed in 10 mL of infusion fluid-based supersaturated calcium phosphate solutions containing FGF-2, as described elsewhere [24, 25, 28, 29].

Briefly, supersaturated calcium phosphate solutions were prepared by mixing clinically available infusions and injection fluids. A Ca solution (Ca^2+^ 8.92 mM) was prepared from Ringer’s solution (Ca^2+^ 2.25 mM) (Otsuka Pharmaceuticals Co., Ltd., Tokyo, Japan) and calcium chloride corrective injection 1 mEq/mL (Ca^2+^ 500 mM) (Otsuka Pharmaceuticals). A phosphate solution (PO_4_^3−^ 2.97 mM) was prepared from Klinisalz® (PO_4_^3−^ 10 mM) (I’rom Pharmaceuticals Co., Ltd., Tokyo, Japan) and dipotassium phosphate corrective injection (1 mEq/mL, PO_4_^3−^ 500 mM) (Otsuka Pharmaceuticals). An FGF-2 solution (100 μg/mL) was prepared by dissolving FGF-2 (Fiblast®; Kaken Pharmaceutical Co., Ltd., Tokyo, Japan) in the Ca solution. Meylon® (NaHCO_3_ 833 mM) (Otsuka Pharmaceuticals) was used as an alkalizer. Supersaturated calcium phosphate solutions containing FGF-2 (0, 0.5, 1.0, or 2.0 μg/mL) were prepared from these four solutions.

The titanium screws were immersed in the supersaturated calcium phosphate solutions containing FGF-2 at 25 °C for 1 day to co-precipitate apatite with FGF-2. The prepared titanium screws coated with FGF-2−apatite composite layers were abbreviated as 25F0, 25F0.5, 25F1, and 25F2.

Another supersaturated calcium phosphate solution containing FGF-2 (4.0 μg/mL) was prepared in the same manner for immersing titanium screws at 37 °C for 2 days [25]. Instead of Meylon, Bifil® (NaHCO_3_ 166 mM) (Ajinomoto Pharmaceuticals Co., Ltd., Tokyo, Japan) was used as an alkalizer. The obtained titanium screws coated with FGF-2−apatite composite layers are abbreviated as 37F4. Uncoated titanium screws are labeled as Ti. The combination of 25F0.5, 25F1, 25F2, and 37F4 is abbreviated as FGF(+), and that of the Ti and 25F0 is abbreviated as FGF(−). The chemical compositions of the supersaturated calcium phosphate solutions are summarized in Table 1.

**Table 1:** Chemical compositions of the supersaturated calcium phosphate solutions

	Titanium screws	
	25F0, 25F0.5, 25F1, 25F2	37F4
Immersing condition		
Temperature	25 °C	37 °C
Duration	1 day	2 days
Ionic concentration (mM)		
Na^+^	147.23	138.87
K^+^	9.92	7.39
Ca^2+^	8.92	3.67
Mg^2+^	0.24	0.22
Cl^−^	153.46	134.39
H_2_PO_4_^-^	0.95	0.90
HPO4^2-^	2.02	0.94
HCO^3−^	15.09	15.09
CH_3_COO^−^	1.90	1.80
Xylitol	31.65	29.93
FGF-2 concentration (μg/mL)	0, 0.5, 1.0, and 2.0	4.0

Characteristics of the FGF-2−apatite composite layers are summarized in Table 2 [24, 29]. The apatite phases of all the layers, which are poorly crystallized, have similar chemical compositions except for the significantly higher Ca/P molar ratio associated with 37F4. The amounts of FGF-2 in the layers obtained by the Bradford method are similar, except for a significantly lower value associated with 25F0.5. The mitogenic activity of the loaded FGF-2 measured by NIH3T3 cell proliferation is also similar, except for a significantly lower value associated with 25F0.5. A typical value of the layer thickness 25F0 was estimated to be 2.9 μm using a CCD laser micrometer.

**Table 2:** Characteristics of the FGF-2-apatite composite layers

	25F0	25F0.5	25F1	25F2	37F4
Ca (μg/screw)	137.7 ± 6.1	114.5 ± 17.2	149.3 ± 22.7	166.2 ± 35.9	143.4 ± 60.9
P (μg/screw)	73.9 ± 3.1	57.3 ± 8.6	73.9 ± 11.0	82.0 ± 17.4	65.2 ± 25.6
Ca/P molar ratio	1.40-1.44	1.55 ± 0.02	1.56 ± 0.01	1.56 ± 0.02	1.67 ± 0.11*
Calcium phosphate phase	Poorly crystallized apatite				
FGF-2 (μg/screw)	–	2.04 ± 1.18*	3.97 ± 1.14	4.62 ± 0.86	4.72 ± 1.91
FGF-2 activity (×10^3^ cells)	–	16.3 ± 2.0*	23.8 ± 4.7	37.2 ± 7.4	31.1 ± 13.2

*p < 0.05

### Animal experiments

All of the inflammation-free histological sections were selected from those obtained previously by percutaneous implantation of 25F0 (*n* = 20), 25F0.5 (*n* = 20), 25F1 (*n* = 20), 25F2 (*n* = 20), 37F4 (*n* = 20), and uncoated titanium (Ti, *n* = 40) screws for 4 weeks in 70 male, 14-week-old Japanese white rabbits (weight range 2.5–3.0 kg) [24, 25, 28]. Wet and rinsed 25F0, 25F0.5, 25F1, 25F2, and 37F4 screws immediately after finishing the immersion were used for the animal experiments. A single physician, who was blinded to the screw identification, performed the operations. After intravenous injection of barbiturate (40 mg/kg body weight), the screws were implanted in both medial proximal tibiae in a direction perpendicular to the tibial shaft axis. First, a small incision (10 mm) was made on the skin, and a perforation 2.5 mm in diameter in both tibial metaphyses. After implantation, the skin was sutured bilaterally to the screw. Postoperatively, each rabbit was allowed to behave freely in its own cage. The rabbits did not receive any antibiotics or treatment for their wounds and were sacrificed 4 weeks after the operation.

The screws were then extracted. The proximal tibial metaphyses were fixed in 10% neutral-buffered formalin for 7 days and then separated into soft-tissue and hard-tissue parts. The hard-tissue parts were decalcified in an ethylenediaminetetraacetic acid solution and embedded in paraffin. The embedded samples were sliced in 5-μm-thick sections that were perpendicular to the tibial longitudinal axis and parallel to the screw hole. The sections were stained with hematoxylin-eosin.

### Histomorphological measurements

The histological sections were randomized and blinded to the screw identification. Two other physicians analyzed the sections independently. Sections with poor conditions as a result of deformation and damage were eliminated from the analysis. A section was considered inflammation-free when both physicians judged that no sign of inflammation was visible. A total of 67 sections from 50 rabbits were identified as inflammation-free. The numbers of inflammation-free sections were 22, 10, 5, 7, 11, and 12 for titanium screws Ti, 25F0, 25F0.5, 25F1, 25F2, and 37F4, respectively.

**Fig. 1:**
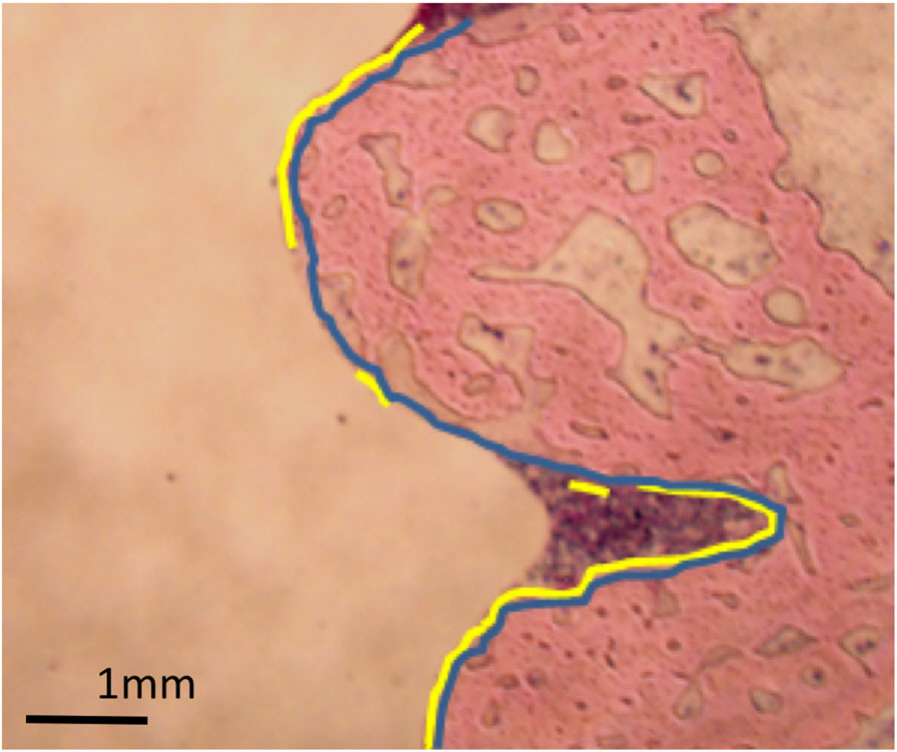
Example of a bone apposition rate measurement. The *yellow* and *blue lines* represent the bone−screw interface line where the bone is in direct contact with the screw and the peripheral line of the screw, respectively

Using the inflammation-free sections, the length of bone−screw interface line where the bone is in direct contact with the screw, the length of the peripheral line of the screw, and the bone apposition to the screw were determined in the cortex and medullary cavity. The bone apposition rate (%) was defined as follows (Fig. 1):

Bone apposition rate (%) = (total length of the bone−screw interface line where the bone is in direct contact with the screw)/(full-length of the peripheral line of the screw in the section).

The length of the bone−screw interface line and the screw’s peripheral line was determined at a magnification of × 12.5 using a Vanox-T microscope (Olympus Optical Co., Ltd., Tokyo, Japan) equipped with a CCD video camera system (DP80; Olympus Optical Co., Ltd., Tokyo, Japan) with the use of Image J software (National Institutes of Health, Bethesda, MD, USA).

### Weibull plot analysis

The bone apposition rate was analyzed using the Weibull plot analysis [27], according to the following Weibull equation:

$$ \mathrm{lnln}\ \left[1/\left(1\hbox{-} \mathrm{S}\right)\right] = \mathrm{m}\  \ln\ \upsigma \hbox{-} \mathrm{m}\  \ln\ \upxi, $$

where *S*, *m*, σ, and ξ indicate the failure probability, Weibull parameter, a measure that gives the failure probability, and a scale parameter, respectively. Thus, the plot of ln σ against lnln [1/(1 − *S*)] gives a straight line with a slope of *m*. In this study, σ is the bone apposition rate, and *S* is the probability of resulting in a bone apposition rate at or less than σ. The measured σ values were arranged in ascending order. S was derived from the average rank method using *S*_*j*_ = *j*/(*N* + 1), where *j* is an order number of an individual σ value, and *N* is the total number of measured σ values.

### Statistical analysis

Student’s *t* test was used to test statistically significant differences in the length of the bone−screw interface line, the screw’s peripheral line, the bone apposition rate and the slope of the regression line of the Weibull plot. The *F* test was used to test a statistically significant difference in dispersion in the frequency histogram. A value of *p* < 0.05 was considered to indicate statistical significance for each analysis.

## Results

**Fig. 2:**
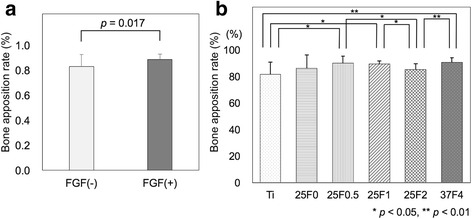
Bone apposition rate for FGF(+) and FGF(−) (**a**), and for titanium screws Ti, 25F0, 25F0.5, 25F1, 25F2, and 37F4 (**b**). The data shown are means ± SD. The combination of 25F0.5, 25F1, 25F2, and 37F4 is abbreviated as FGF(+), and the combination of Ti and 25F0 is abbreviated as FGF(−)

**Fig. 3:**
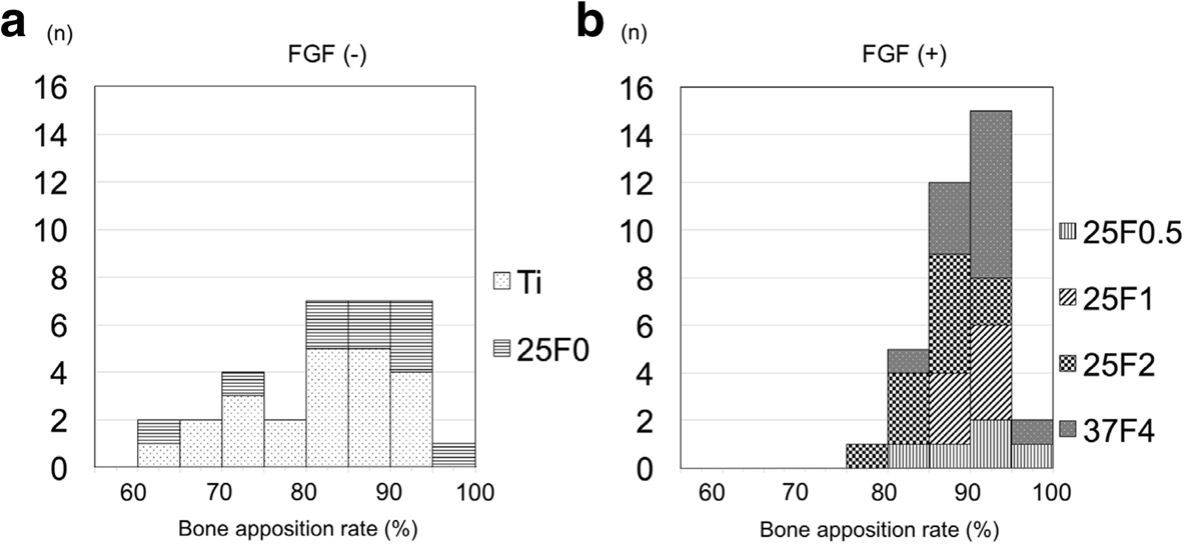
Frequency histograms of bone apposition rates for FGF(−) (**a**) and FGF(+) (**b**). The combination of 25F0.5, 25F1, 25F2, and 37F4 is abbreviated as FGF(+), and the combination of Ti and 25F0 is abbreviated as FGF(−)

The bone apposition rate was significantly higher for FGF(+) (88.6 ± 4.4%) than for FGF(−) (83.0 ± 9.5%) (*p* = 0.017) (Fig. 2). The bone-screw interface line was significantly longer for FGF(+) (43.5 ± 7.8 mm) than for FGF(−) (38.9 ± 9.2 mm) (*p* = 0.03) while there was no significant difference in the length of screw’s peripheral line between FGF(+) (49.2 ± 9.4 mm) and FGF(−) (46.8 ± 10.1 mm) (*p* = 0.30) (Table 3). The frequency distribution of the bone apposition rate for FGF(+) was very narrow and nearly symmetrical, with all of the values being >75% (Fig. 3). In contrast, the frequency distribution of the bone apposition rate for FGF(−) was broad and apparently asymmetrical, with one-fourth of the values being <75%. The frequency distribution was significantly broader for FGF(−) than for FGF(+) (*p* < 0.0001). Thus, qualitatively, the FGF(+) group resulted in a lower incidence of impaired bone formation around screws than the FGF(−) group.

**Table 3:** Lengths of bone-screw interface line and screw’s peripheral line in FGF(−) and FGF(+) groups

	FGF(−) (mm)	FGF(+) (mm)	*P* value*
Bone-screw interface line	38.9 ± 9.2	43.5 ± 7.8	0.03
Screw’s peripheral line	46.8 ± 10.1	49.2 ± 9.4	0.30

* Student’s *t* test

**Fig. 4:**
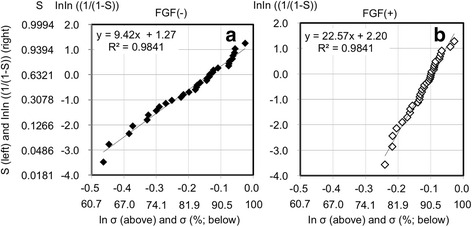
Weibull plots of the bone apposition rates for FGF(−) (**a**) and FGF(+) (**b**) that show the probability (*S*) of resulting in a bone apposition rate in the range of 0 to σ (%). The *y* and *x* of the regression lines represent lnln [1/(1 − *S*)] and ln σ, respectively. The combination of 25F0.5, 25F1, 25F2, and 37F4 is abbreviated as FGF(+), and the combination of Ti and 25F0 is abbreviated as FGF(−)

We used Weibull plot analysis to analyze the risk of impaired bone formation quantitatively (Fig. 4). The Weibull plot was linear for both FGF(+) and FGF(−). The slope of the regression line was significantly higher for FGF(+) (22.6) than for FGF(−) (9.42) (*p* = 7.3 × 10^−34^). Using the regression lines, the risk of impaired bone formation was calculated at some threshold vales of bone apposition rate which were selected in an arbitrary manner (Table 4). For example, if impaired bone formation is defined as a state at ≤63.75% of the bone apposition rate, the probabilities of impaired bone formation are 3.5 × 10^−4^ and 0.05 for FGF(+) and FGF(−), respectively. In the same manner, if impaired bone formation is defined as a state at ≤68.82% of the bone apposition rate, the probabilities of impaired bone formation are 0.002 and 0.1 for FGF(+) and FGF(−), respectively. Thus, the risk of impaired bone formation is remarkably lower for FGF(+) than for FGF(−).

**Table 4:** Probability of impaired bone formation in different setting of threshold of bone apposition rate

Threshhold setting in bone apposition rate	Probability of impaired bone formation	
	FGF(−)	FGF(+)
≤68.82%	0.1	0.002
≤63.75%	0.05	3.5 × 10^−4^
≤50%	0.005	1.4 × 10^−6^

## Discussion

FGF(+) had a significantly higher bone apposition rate and lower incidence of impaired bone formation around the screws than FGF(−) in inflammation-free histological sections obtained by percutaneous implantation of external fixation screws. Because there were individual differences in bone-forming ability among the animals with an implanted screw, we recorded and compared the ranges of the bone apposition rates for the FGF(+) and FGF(−) groups (Fig. 3).

The average bone apposition rate was only slightly higher for FGF(+) than for FGF(−), with significant difference (*p* = 0.017) (Fig. 2a). The difference in the frequency distribution of the bone apposition rate, however, was more marked and significant between the FGF(+) and FGF(−) groups (Fig. 3). The frequency distribution for FGF(+) was very narrow, whereas that for FGF(−) was broad. The broader distribution for FGF(−) was a result of the presence of low values of the bone apposition rate, which caused an asymmetrical frequency distribution.

The sharp contrast in the broadness of the frequency distribution suggested that a small number of animals with less-strong bone-forming ability were randomly assigned to both the FGF(+) and FGF(−) groups. In addition, FGF-2 enhanced the bone formation of only those animals with less-strong bone-forming ability in the FGF(+) group. FGF-2 is known to exhibit a bell-shaped dose response, where an excess of FGF-2 has less or no enhancement effect on bone formation [10, 14]. Animals having a sufficient level of endogenous FGF-2 in the FGF(+) group would not have responded to exogenous FGF-2. As a result, there was a lower incidence of impaired bone formation around the FGF(+) screw than the FGF(−) screw.

**Fig. 5:**
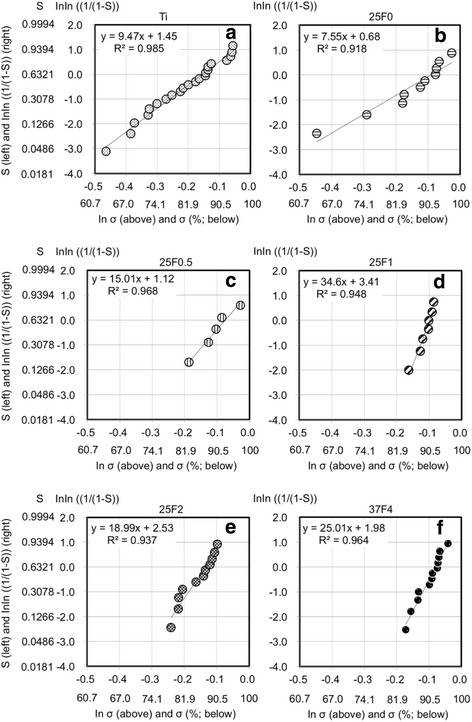
Weibull plots of the bone apposition rates for the six titanium screw groups. Weibull plots of bone apposition rate for Ti (**a**), 25F0 (**b**), 25F0.5 (**c**), 25F1 (**d**), 25F2 (**e**), and 37F4 (**f**) show the probabilities (S) of resulting in a bone apposition rate in the range of 0 to σ (%). The *y* and *x* of regression lines represent lnln [1/(1 − *S*)] and ln σ, respectively

The risk of impaired bone formation was remarkably lower for FGF(+) than for FGF(−), as revealed by the Weibull plot analysis (Table 4). The Weibull plot was linear for both FGF(+) and FGF(−). Thus, the present analysis meets the theoretical prerequisites of the Weibull plot analysis. The fact that the slope of the regression line was noticeably greater for FGF(+) than for FGF(−) proved that there is significantly less risk of impaired bone formation around the screw with the FGF(+) condition than that with the FGF(−) condition. The risk is expressed as a probability (*S*) of resulting in a bone apposition rate equal to or less than a specific value of σ. For example, if the specific values are set at 68.82, 63.75, and 50%, the probabilities of resulting in a bone apposition rate equal to or less than 68.82, 63.75, and 50% are 0.1, 0.05, and 0.005, respectively, for FGF(−). For FGF(+), the probabilities for the same specific values are 0.002, 3.5 × 10^−4^, and 1.4 × 10^−6^ (Table 4). Note that the difference between FGF(−) and FGF(+) in terms of the risk probabilities (e.g., 0.1:0.002 at 68.82%, 0.05:3.5 × 10^−4^ at 63.75%, and 0.005:1.4 × 10^−6^ at 50%) increases when the specific value of the bone apposition rate decreases. The thresholds of ≤68.82%, ≤63.75%, and ≤50% correspond to bottom 0.5%, 5%, and 10%, respectively, of FGF(−) samples in the rank of bone apposition rate. Thus, FGF(+) was more reliable than FGF(−) in terms of preventing impaired bone apposition regardless of the individual’s bone-forming ability. The decreased risk is not a result of the apatite, but rather the presence of FGF-2 in the FGF-2−apatite composite layer, as the slopes for Ti and 25F0 (apatite-coated Ti) were similar and significantly lower than those for 25F0.5, 25F1, 25F2, and 37F4 (Fig. 5).

It is feasible to apply the FGF-2−apatite composite layer to internal fixation screws, which have varied stability at the screw–bone interface depending on the quantity, quality, and regeneration of bone surrounding the screw [30–32]. The stability of the screw−bone interface is characterized by the extraction torque and pull-out resistance [33]. There was higher fixation strength of the screws coated with hydroxyapatite than the uncoated screws owing to direct bone formation on hydroxyapatite [34]. Hydroxyapatite coated with an FGF-2−apatite composite layer at an optimized dose produced more bone formation than that with uncoated hydroxyapatite [23]. Enhanced bone formation associated with other FGF-2-coated implants resulted in greater mechanical stability at the implant–bone interface [35, 36]. In the present study, the screws coated with an FGF-2−apatite composite layer showed significantly less risk of impaired bone formation than the uncoated and apatite-coated screws. Such a decreased risk of impaired bone formation would result in a decrease in the risk of impaired pull-out resistance. Thus, use of an FGF-2−apatite composite layer to coat internal fixation screws is feasible in that it could decrease the risk of screw loosening. This characteristic is clinically useful because screw loosening leads to severe complications.

In the present study, the use of the Weibull plot analysis provided new insights into the effect of FGF-2 on bone formation. The previously reported effects of FGF-2 on increasing bone formation were equivocal. Some authors reported increased effects, whereas others reported no effect or only a limited effect [37–40]. All of these studies compared the average amount of bone formation or osteogenic differentiation. As shown in Fig. 2, a comparison of the average values is not efficient for detecting the difference in frequency of outlier values when the frequency is low. The Weibull plot analysis enabled such low probability events to be detected. As a result, it revealed a restorative effect of FGF-2 on the impaired bone-forming ability. However, the mechanism that underlies the restoring effect remains unclear.

Limitations of the present study are that (i) the FGF-2−apatite composite layers that were prepared under different conditions are involved in the analysis, (ii) the number of inflammation-free histological sections in each group was small and varied greatly, and (iii) no pull-out strength data are presented.

## Conclusions

Histomorphometrical analysis of inflammation-free histological sections obtained using percutaneous implantation of external fixation screws revealed that, on average, screws coated with FGF-2−apatite composite layers showed a slightly higher bone apposition rate, with a significant difference from the uncoated and apatite-coated screws. However, FGF-2−apatite composite layers have a much more marked effect on reducing the incidence of impaired bone apposition than on enhancing the bone apposition rate. The Weibull plot analysis revealed that the risk of resulting in less than 63.75%, for example, in bone apposition rate was reduced by 1/142 times (3.5 × 10^−4^/0.05) with the use of FGF−apatite composite layer coating compared with uncoated and apatite-coated screws. Therefore, FGF-2−apatite composite layers are feasible for coating internal fixation screws, as it is known that bone quality, quantity, and formation around the screw is crucial to prevent loosening of internal fixation screws. Further study is required.

## Future study

Animal studies that include implanting internal fixation screws, measuring local bone formation, and determining the pull-out strength of the screw are required in combination with the Weibull plot analysis. These studies could further clarify whether the FGF-2−apatite composite layers reduce the risk of screw loosening.

## Abbreviations

BMP: Bone morphogenetic proteins
FGF-2: Fibroblast growth factor-2
FGF-2−apatite: Fibroblast growth factor-2−apatite
